# Identification of Factors Affecting Predation Risk for Juvenile Turtles Using 3D Printed Models

**DOI:** 10.3390/ani10020275

**Published:** 2020-02-11

**Authors:** Sasha J. Tetzlaff, Alondra Estrada, Brett A. DeGregorio, Jinelle H. Sperry

**Affiliations:** 1 Department of Natural Resources and Environmental Sciences, University of Illinois at Urbana-Champaign, Urbana, IL 61801, USA; 2 U.S. Army Construction Engineering Research Laboratory, Champaign, IL 61822, USA; 3 U.S. Geological Survey, University of Arkansas Fish and Wildlife Cooperative Research Unit, Fayetteville, AR 72701, USA

**Keywords:** habitat selection, motion-triggered camera, predator-prey ecology, sensory cue, *Terrapene carolina*, three-dimensional printing

## Abstract

**Simple Summary:**

Turtles are one of the most threatened vertebrate groups. High rates of juvenile predation may contribute to population declines for many species, but predator identities and factors contributing to predation risk for juveniles are largely unknown due to their highly secretive nature. Alternatives to studying live juveniles are needed. By monitoring three-dimensional printed models resembling juvenile box turtles (*Terrapene carolina*) with motion-triggered cameras in the Midwestern USA, we found raccoons (*Procyon lotor*) were the dominant predator that interacted with models, followed by rodents (Sciuridae). Mesopredator interactions with models were less likely in habitats with higher vegetative ground cover. However, availability of sensory cues (visual or olfactory) provided by models did not influence mesopredator interactions, suggesting they used multiple senses to detect models. Rodents interacted with models that were closer to woody structure, likely because they commonly utilize such small-scale habitat features. Rodents interacted with exposed models more than concealed models, reflecting their predominantly visual foraging behavior. Overall, our results suggest juvenile turtle habitat selection could affect predator-specific predation risk but behavioral differences between particular predators are also important determinants of risk. These findings have implications for management efforts aimed at reducing encounters between juvenile turtles and their major predators.

**Abstract:**

Although it is widely accepted that juvenile turtles experience high levels of predation, such events are rarely observed, providing limited evidence regarding predator identities and how juvenile habitat selection and availability of sensory cues to predators affects predation risk. We placed three-dimensional printed models resembling juvenile box turtles (*Terrapene carolina*) across habitats commonly utilized by the species at three sites within their geographical range and monitored models with motion-triggered cameras. To explore how the presence or absence of visual and olfactory cues affected predator interactions with models, we employed a factorial design where models were either exposed or concealed and either did or did not have juvenile box turtle scent applied on them. Predators interacted with 18% of models during field trials. Nearly all interactions were by mesopredators (57%) and rodents (37%). Mesopredators were more likely to attack models than rodents; most (76%) attacks occurred by raccoons (*Procyon lotor*). Interactions by mesopredators were more likely to occur in wetlands than edges, and greater in edges than grasslands. Mesopredators were less likely to interact with models as surrounding vegetation height increased. Rodents were more likely to interact with models that were closer to woody structure and interacted with exposed models more than concealed ones, but model exposure had no effect on interactions by mesopredators. Scent treatment appeared to have no influence on interactions by either predator group. Our results suggest raccoons can pose high predation risk for juvenile turtles (although rodents could also be important predators) and habitat features at multiple spatial scales affect predator-specific predation risk. Factors affecting predation risk for juveniles are important to consider in management actions such as habitat alteration, translocation, or predator control.

## 1. Introduction

Turtles (order Testudines) are one of the most imperiled vertebrate groups; approximately 60% of species are threatened with extinction or have recently become extinct [1]. High nest and juvenile predation are thought to be major threats to population viability [2,3]. Predator identities and factors contributing to predation risk have been documented for nests [4] and adults [5], but comparatively little is known for juveniles. Therefore, conservation management efforts for turtles could benefit from a greater understanding of relationships between juveniles and their predators [6].

Limited available evidence suggests mammalian carnivores, particularly mesopredators [3,7], and predatory birds [8,9,10] are the most common predators of juvenile terrestrial turtles. Small mammals such as Sciurid rodents also have been implicated with depredating juveniles [11,12,13]. Juvenile turtles have lower estimated survival than adults [14], likely in part due to increased vulnerability to predators resulting from smaller body sizes and incomplete hardening of the shell [15]. Their primary anti-predator strategy appears to entail habitat selection facilitating concealment such as burrowing in substrate or seeking cover (e.g., under woody structure, leaf litter, or dense vegetation) [16]. However, it is not well understood how this mitigates predation risk, especially considering risk is often temporally and spatially variable based on predator groups present [17,18,19]. Additionally, the various sensory modalities used by foraging predators could differentially affect risk for juvenile turtles. For example, many birds [20,21] and rodents [22,23] are primarily visual foragers, whereas olfaction typically plays a large role in prey detection for meso-mammals [24,25]. Obtaining sufficient numbers of wild-born juveniles to investigate predator-prey relationships has proved to be very challenging due to their highly elusive behavior, which is common for many reptiles [26]. Alternatives to studying live turtles are thus needed.

Physical models of focal organisms are commonly used to study predator-prey interactions. These studies have traditionally relied on models constructed from clay (plasticine) or silicone and have been useful for understanding factors contributing to predation risk [27]. Advancements in three-dimensional (3D) printing technology has facilitated use of more realistic and standardization of models for studying the ecology of numerous species [28,29]. In addition to identification of predators, prey models can be used to understand timing of predation (particularly when monitored with cameras), variation in predation risk based on biotic or abiotic factors, and mechanisms of prey detection [30]. For instance, visual and olfactory cues can be experimentally manipulated to determine their importance on predator-specific interactions with models [31,32].

We sought to gain a better understanding of factors influencing predation risk for juvenile terrestrial turtles using 3D printed models resembling eastern box turtles (*Terrapene carolina*). This species occurs over much of the eastern United States and is of conservation concern in part due to intense nest and juvenile predation [33]. As such, eastern box turtles are listed as Vulnerable by the IUCN and included in CITES Appendix II [33]. They are typically associated with forested habitats but also occupy forest edges, shallow wetlands, and grasslands such as old field and prairie ([2] and references therein). We monitored models across these habitats with motion-triggered cameras at three sites within the geographical range of eastern box turtles. We employed a factorial design to explore 1) potential juvenile box turtle predator identities, 2) if predation risk varied across habitat types and in relation to microhabitat features, and 3) how the presence or absence of visual (exposed or concealed) and olfactory cues (with or without juvenile box turtle scent) solicited predator-specific interactions (detections and attacks) with 3D turtle models.

## 2. Materials and Methods

### 2.1. Turtle Models 

**Figure 1:**
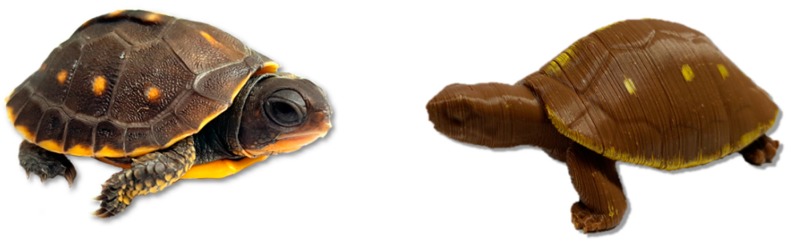
Juvenile eastern box turtle (*Terrapene carolina*) in comparison to a three-dimensional printed turtle model. Photos by Sasha Tetzlaff.

The design for our 3D models (Figure 1) was sourced from an existing .stl file (https://www.thingiverse.com/thing:182332). Models were printed using 2.85 mm brown PLA filament (Shenzhen Esun Industrial Co., Ltd., Shenzhen, China) in an Ultimaker 2 + 3D printer (Ultimaker B.V., Geldermalsen, The Netherlands). Each model weighed 21 g and was 5 cm long. Growth rate and age-specific body size data for wild juvenile eastern box turtles are scarce compared to adults and likely vary according to factors such as latitude and resource availability, but we suspect the length of our models represents the approximate length of young (<3 year-old) juveniles in many populations [2,34]. We applied conservative yellow markings resembling the pattern of a juvenile box turtle’s carapace on each model with a paint pen.

### 2.2. Study Sites

**Figure 2:**
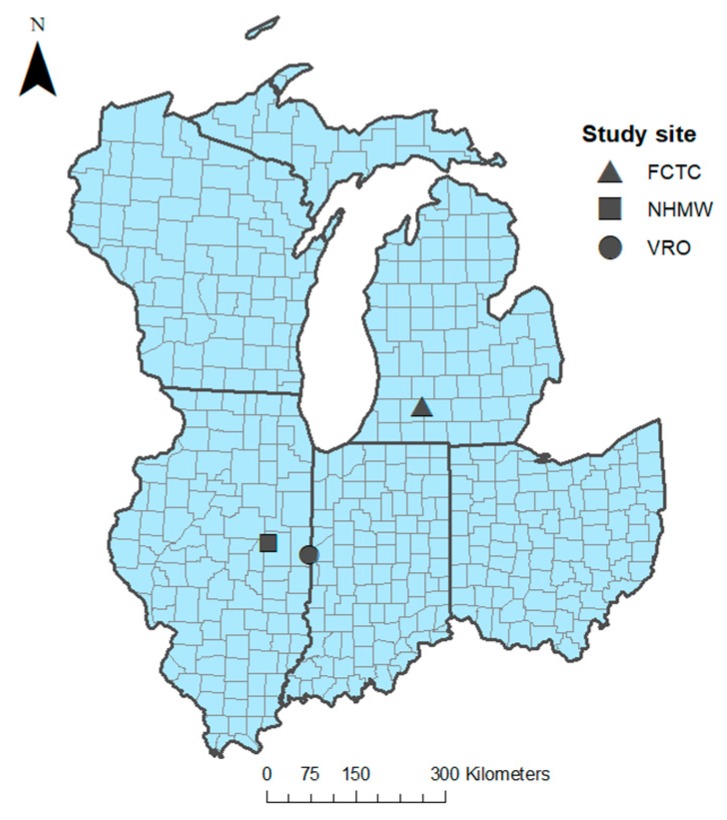
Study sites at Fort Custer Training Center (FCTC), Michigan, and Nettie Hart Memorial Woods (NHMW) and Vermilion River Observatory (VRO), Illinois, USA.

This research took place in 2018 at three study sites: one in southwestern Michigan and two in east-central Illinois, USA (Figure 2). Fort Custer Training Center (hereafter FCTC) is an Army National Guard training facility located in Kalamazoo and Calhoun counties, Michigan. The installation has approximately 3000 ha of natural habitat comprised primarily of woodlands (2023 ha), wetlands (485 ha), and old field and prairie (485 ha). 

Located in Vermilion County, Illinois, Vermilion River Observatory (hereafter VRO) is an approximately 200 ha site comprised mostly of deciduous forest (112 ha) but also contains roughly 36 ha of old fields and successional habitat as well as 45 ha of agricultural land. Nettie Hart Memorial Woods (hereafter NHMW) is a 16 ha property located in Champaign County, Illinois that is comprised mostly of secondary growth upland dry-mesic woodlands. The Sangamon River runs along the western edge of the property and a permanent creek (4 m average bank width) runs along the south edge. The lowlands are seasonally flooded near waterways. Agricultural land and housing developments surround this site. The three study sites were largely chosen because they are closed to the public; also, research on juvenile box turtles was concurrently being conducted at FCTC [35]. Box turtles are known to occur at FCTC and VRO but to our knowledge have not been detected at NHMW; we encountered none while conducting fieldwork at NHMW. Whether factors such as abundances and activity patterns of predators vary between our study sites is unknown, but the observed predator species were very similar among sites.

### 2.3. Field Trials

We conducted 372 trials (unique model placements): 208 at FCTC, 72 at NHMW, and 92 at VRO. We conducted field trials from May to August as this is the general timeframe when eastern box turtles are most active (i.e., not belowground overwintering) and thus most likely susceptible to predation [2]. Our general approach was to place sets of models across habitat types commonly utilized by box turtles at each site: forest, wetland, and grassland. Due to availability of habitats and site accessibility at each study area, we placed models in forest, wetland, and grassland at FCTC; forest and grassland at VRO; and forest and wetland at NHMW. We also placed models in forest edges at each site, defined as an approximate 15 m boundary between forest and other habitat types [36]. Each time a model was placed, we measured several microhabitat variables that we *a priori* predicted would influence predation risk. We measured distance (cm) to nearest woody structure (logs, stumps, and piled woody debris) up to 10 m from a model as several species of rodents are known to use these objects as cover [37]. Because vegetation density and height influence visibility, we also measured modal and maximum height (cm) of understory (≤250 cm) vegetation surrounding or nearest to models (generally within 1 m).

We used a factorial design to test the importance of and interaction between model concealment and scent on turtle models. We placed four models in each habitat type at each site during a field trial. Two models were mostly exposed (~85% visible as viewed from directly above the model) and two were mostly concealed (~15% visible as viewed from directly above the model). Concealed models were partially buried in soil and/or covered by leaf litter, woody debris, and live vegetation. Two of the four models were also “scented” with olfactory cues from eastern box turtles. We used a colony of 20 captive-born juvenile eastern box turtles [35] as an odorant source for scented models. We collected olfactory cues by swabbing the shell and skin of juveniles with sterile cotton-tipped applicators. We placed applicator tips in 10 mL headspace vials, and vials were stored at −20 °C until the day a swab was used in a trial. We also collected and stored water that juveniles had soaked in for at least 24 h in their permanent enclosures to place on models when used in a trial. We put scented swab tips underneath the front end of models when used in trials so the swab was somewhat concealed. However, we still aimed for the swab to be subjected to airflow, allowing detection by olfactory-hunting predators [24]. We also poured approximately 30 mL of water collected from captive juvenile turtles’ cages on each scented turtle. We poured an equivalent amount of distilled water on unscented models as a liquid control. As an additional control, we also placed unscented swab tips under unscented models in the same fashion as for scented models. Details of how we quantified chemical compounds on swabs (scented and unscented) are described in Appendix A.

We monitored models with game cameras (Bushnell^®^ Trophy Cam HD, Bushnell Outdoor Products, Overland Park, KS, USA) mounted to 120 cm tall metal stakes placed 1 m from models. Cameras were programmed to record a 15–30-sec video once motion-triggered. We were constrained by the number of available cameras, so we monitored models in pairs in each habitat type. Each of the paired models were 60 cm apart. Each model in a pair was either exposed or concealed. Scented models were always paired together so that the availability of olfactory cues would not confound detection of an unscented model. We randomly determined the direction and distance model pairs were spaced apart within a habitat patch. We wore nitrile gloves when handling all relevant supplies and replaced gloves when switching from handling scented to unscented models. Models that were scented were never used in the unscented treatment and vice versa. The person placing models in the field recorded a video clip at the beginning of a trial pointing out where models were placed to facilitate review of camera media when scoring predator interactions with models. Similar to previous studies [31,38], trials lasted for five days, and we used new locations to place models at the start of each trial. We washed models with warm water and soap between trials, but models that were attacked by predators were replaced with a model that had not yet been used in a trial.

When reviewing camera media, we classified predator interactions with models similar to previous work [30]. We defined an “attack” as when a predator bit or grasped a model. “Detections” were defined as when a predator clearly noticed a model by altering movement and directing attention to it with its eyes or nose but did not attack. We classified models as having “no interaction” if we did not identify detections of or attacks on a model during a trial. Although video footage revealed a given model was interacted with more than once during a trial on numerous occasions, we used only the first detected interaction from the beginning of a trial in analyses. This reduced potentially confounding effects of multiple interactions with a model due to behavior unrelated to predation. For example, we observed instances of multiple raccoons (*Procyon lotor*) urinating on a model after it had been interacted with by a conspecific.

### 2.4. Data Analyses

We conducted all statistical analyses in R version 3.4 [39]. To evaluate the effects of numerous predictor variables on a model turtle’s fate during a trial, we used multinomial logistic regression (multinom function in the nnet package, [40]). Small sample sizes of interactions by predator species required that we grouped them based on ecological similarity. Multinomial regression models thus consisted of three response variables: interaction by mesopredator, interaction by rodent, or no interaction. The “mesopredator” category included interactions by raccoons and Virginia opossums (*Didelphis virginiana*). The “rodent” category included interactions by eastern chipmunks (*Tamias striatus*), eastern fox squirrels (*Sciurus niger*), and eastern grey squirrels (*Sciurus carolinensis*). Our reference category was “no interaction”.

Our predictor variables in a global model included study site, habitat type, scent treatment, exposure treatment, modal and maximum vegetation height, and distance to woody structure. We included an interaction term for scent and exposure treatment but analyzed other variables as additive effects. We assessed multinomial models for all possible combinations of predictors as well as an intercept-only model using the dredge function in the MuMIn package [41]. Continuous predictors were not highly correlated (|r| < 0.70) with one another, so we allowed inclusion of any predictor in a given model. We used Akaike’s Information Criterion [42] corrected for small sample size (AICc) to evaluate model support and calculate model weights (i.e., relative model importance). If no single model was strongly supported (i.e., garnered most of the Akaike weight), we used multimodel inference and generated model-averaged parameter estimates from the top-ranked models summing to 95% of the total Akaike weight in the candidate set [43]. We generated odds ratios from parameter estimates and considered those for which the 85% confidence interval (CI) (as suggested by [44]) did not overlap one to be most informative. Collectively, this helped us identify more meaningful parameters from less informative ones in a large candidate model set [44].

To examine if predator groups differed in how they interacted with models (i.e., if mesopredators were more likely to attack models than rodents), we used binomial logistic regression. Interaction strength (“detect” or “attack”) was the response variable, and predator group (mesopredator or rodent) was the predictor. “Detect” was the reference variable, meaning we were predicting the probability of attack. 

## 3. Results

### 3.1. Descriptive Results

**Figure 3:**
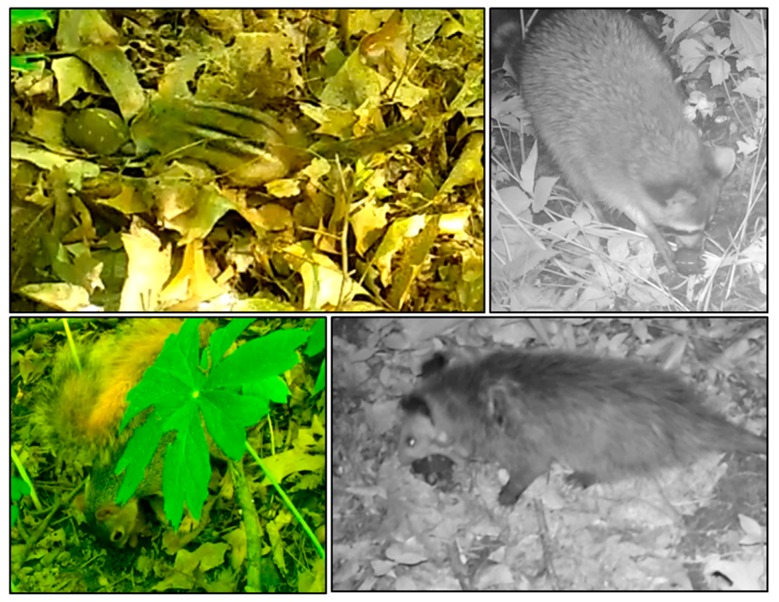
Still images from trail camera videos showing interactions with three-dimensional printed turtle models by mammalian predators. Clockwise from top-left: eastern chipmunk (*Tamias striatus*), raccoon (*Procyon lotor*), Virginia opossum (*Didelphis virginiana*), and eastern fox squirrel (*Sciurus niger*).

**Figure 4:**
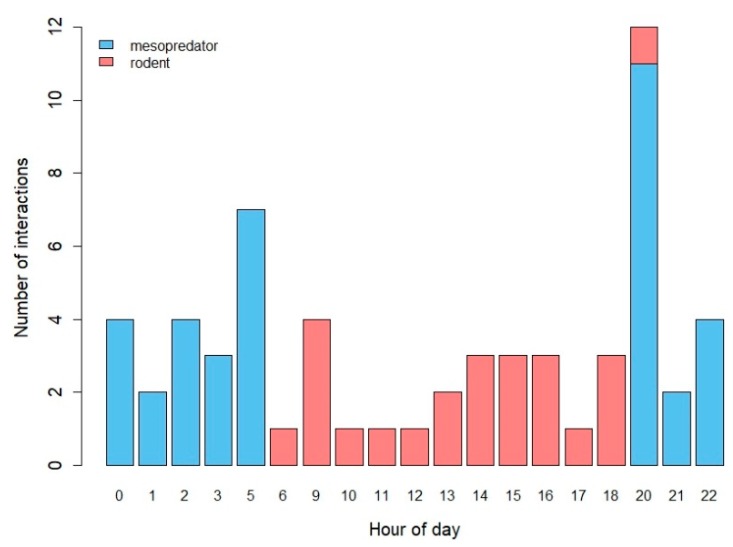
Number of interactions by mesopredators and rodents with three-dimensional printed turtle models by hour of day. Note some hours of day are missing due to no observed interactions during these times.

We documented 65 interactions (18% of trials) with the models. Nearly all interactions were by small- and medium-sized mammals (Table 1; Figure 3; Videos S1–S9). Most interactions were by raccoons (n = 33 interactions, 51%), followed by rodents (n = 24 interactions, 37%). Models were attacked 29 times (45% of interactions, 8% of all trials) and detected 36 times (55% of interactions, 10% of all trials). Raccoons attacked models the most (n = 22 attacks, 76%), whereas eastern chipmunks detected models the most (n = 15 detections, 42%). Two interactions each by wild turkey (*Meleagris gallopavo*) (Video S10) and an unidentified small mammal species (collectively 6% of interactions) were not included in analysis due to the small sample sizes. We also observed a woodchuck (*Marmota monax*) biting a model but did not include this in analysis due to the single instance and because interactions with the same model by several raccoons occurred earlier in the trial. Most interactions by mesopredators occurred from evening to early morning hours (20:00–05:00), when box turtles are generally not active [2,45]. Rodents interacted with models primarily during early morning to early evening hours (06:00 h–18:00 h), which aligns with the typical diurnal activity of box turtles [2] (Figure 4).

### 3.2. Mesopredator Interactions

**Figure 5:**
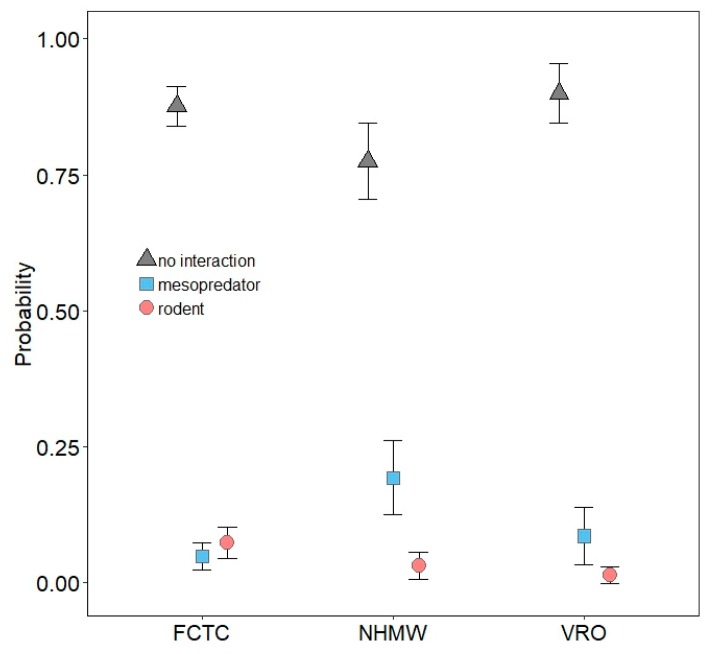
Probability with 85% confidence interval of mesopredators and rodents interacting with three-dimensional printed turtle models (or no interaction) at three study sites: Fort Custer Training Center (FCTC), Michigan, and Nettie Hart Memorial Woods (NHMW) and Vermilion River Observatory (VRO), Illinois, USA.

**Figure 6:**
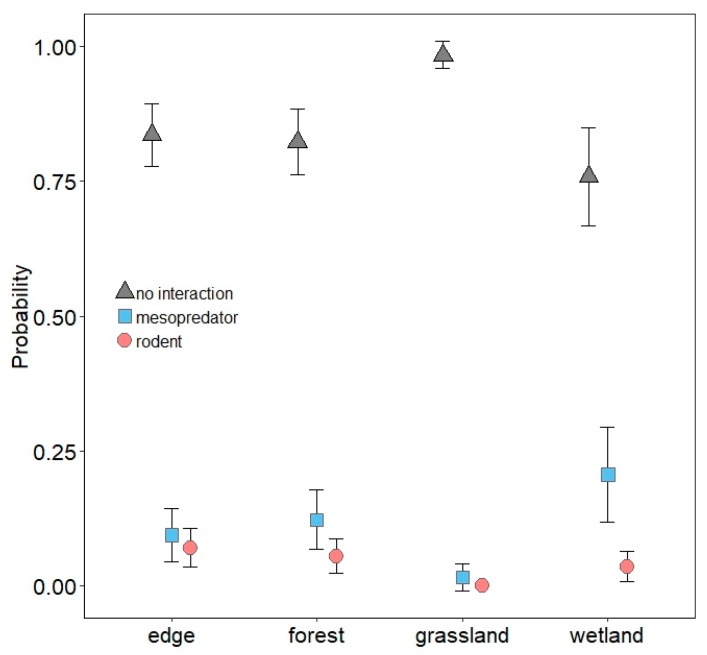
Probability with 85% confidence interval of mesopredators and rodents interacting with three-dimensional printed turtle models (or no interaction) in forest edges, forests, grasslands, and wetlands.

**Figure 7:**
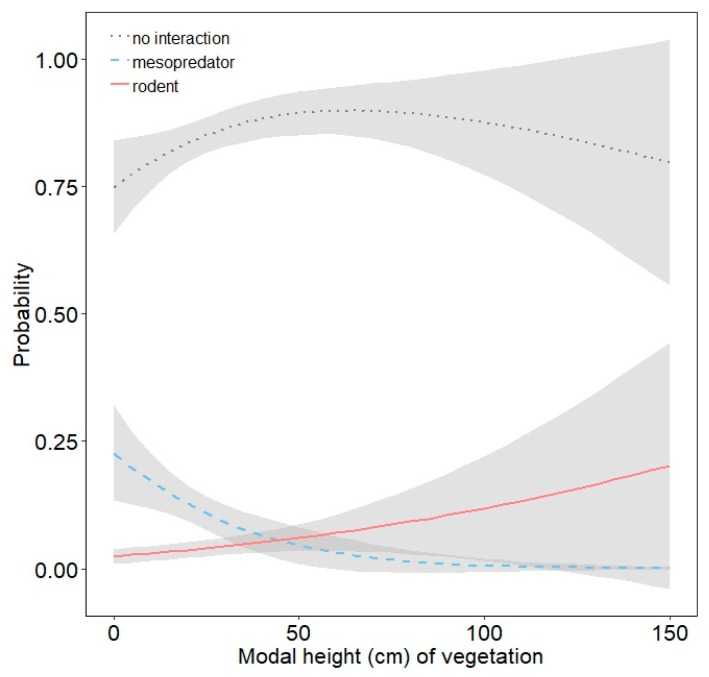
Probability with 85% confidence interval of mesopredators and rodents interacting with three-dimensional printed turtle models (or no interaction) as a function of modal vegetation height (cm) around models.

**Figure 8:**
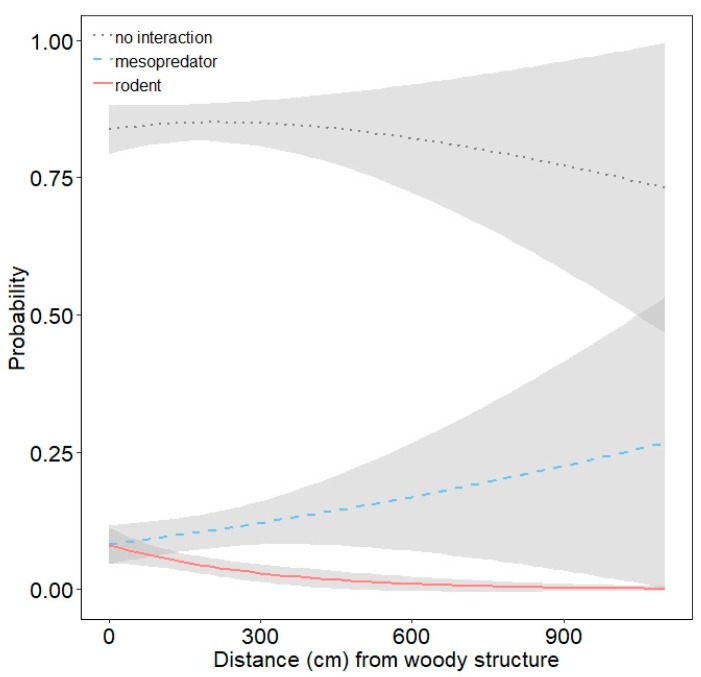
Probability with 85% confidence interval of mesopredators and rodents interacting with three-dimensional printed turtle models (or no interaction) as a function of distance (cm) of models from woody structure.

**Figure 9:**
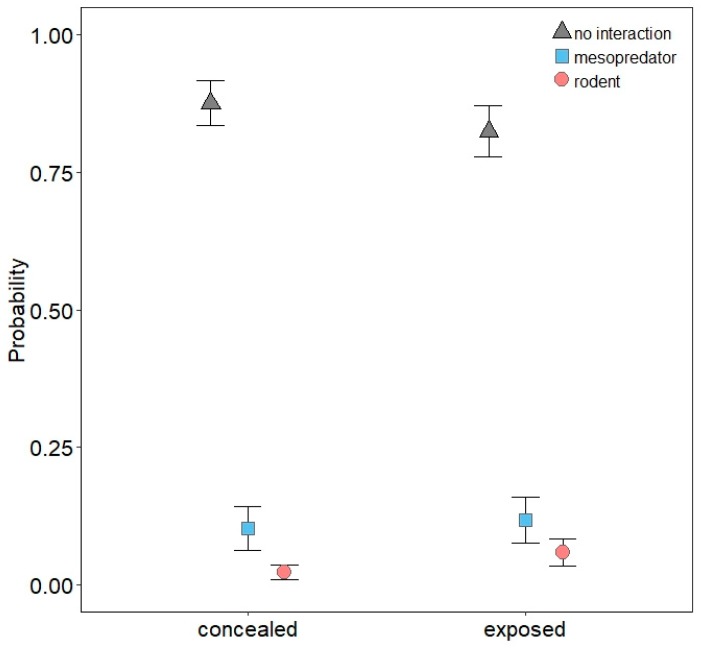
Probability with 85% confidence interval of mesopredators and rodents interacting with three-dimensional printed turtle models (or no interaction) depending on whether models were exposed or concealed.

**Figure 10:**
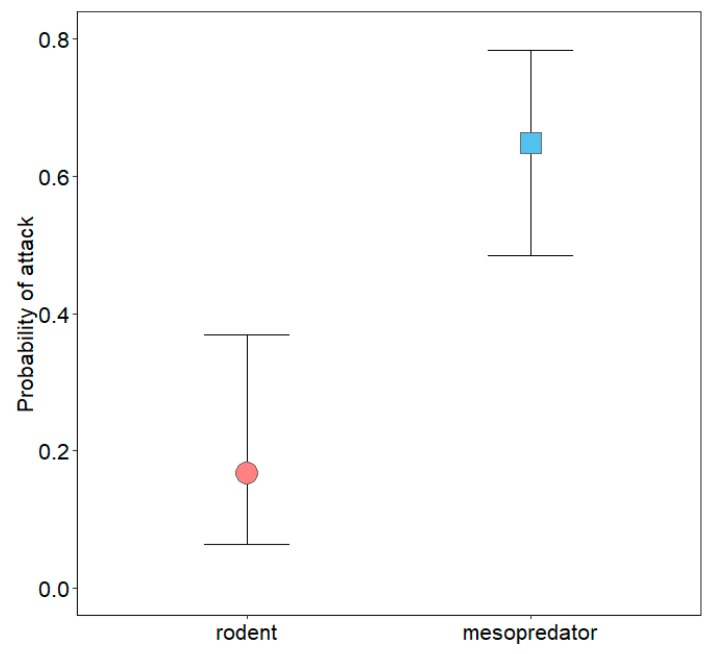
Probability with 95% confidence interval of rodents and mesopredators attacking three-dimensional printed turtle models.

No multinomial regression model emerged as being strongly supported, as the top-ranked model garnered only 23% of the weight of evidence (Table S1). Odds ratios from model-averaged parameter estimates for each predictor are presented in Table 2. Mesopredators were nearly five times more likely to interact with models at NHMW than FCTC, but interactions between FCTC and VRO were similar (Table 2; Figure 5). Interactions by mesopredators were more likely in wetlands than edges and greater in edges than grasslands, but interaction probabilities in forest and edges were similar (Table 2; Figure 6). Mesopredators were less likely to interact with models as modal vegetation height around models increased (Table 2; Figure 7). Effect sizes for maximum vegetation height and distance from woody structure were similar to that for modal vegetation height, but confidence intervals for their estimates slightly overlapped one (Table 2) and had less effects on interaction probabilities (Figure 8, Figure S1). Exposed or scented models were no more likely to be interacted with by mesopredators than concealed or unscented models, respectively (Table 2; Figure 9, Figure S2). Mesopredators were 9.23 times (95% CI for the odds ratio: 2.81 to 37.18) more likely to attack models than rodents (Figure 10).

### 3.3. Rodent Interactions

Rodents were less likely to interact with models at VRO than FCTC, but interactions between FCTC and NHMW were similar (Table 2; Figure 5). Habitat type had little influence on rodent interactions (Table 2; Figure 6). Interactions by rodents were less likely as models were farther from woody structure (Table 2; Figure 8). Effect sizes for modal and maximum vegetation height were similar to that for distance from woody structure, but confidence intervals for their estimates slightly overlapped one (Table 2) and had less effects on interaction probabilities (Figure 7, Figure S1). Rodents were more likely to interact with exposed models than concealed ones (Table 2; Figure 9), but scent treatment had no discernable effect on interaction probabilities (Table 2; Figure S2).

## 4. Discussion

Our results corroborate claims suggesting raccoons are a primary predator of North American juvenile turtles [3], including box turtles [2]. Habitat type appeared to most influence raccoon interactions. Raccoons use a variety of habitats [46,47,48,49], including those used by eastern box turtles [2]. We found the probability of mesopredator interactions with models was highest in wetlands. Many of the wetland habitats we placed models in were adjacent to linear corridors mesopredators are known to traverse [50,51], thus potentially increasing the likelihood models would be detected in these areas. For example, more than half (58%) of mesopredator interactions at NHMW occurred in river floodplain. Edges have been reported as risky habitats for reptiles [31,52], but we noted intermediate levels of interactions in edges compared to other habitat types. Mesopredator interactions were lowest in grasslands, and dense ground vegetation in these habitats could have provided visual [38] and olfactory (via disruption of airflow, [25]) concealment. This is supported by our finding that mesopredators were less likely to interact with models as vegetation height surrounding them increased. However, we did not find availability of scent or visual cues generally influenced mesopredator interactions with models, which likely reflects the variety of sensory cues raccoons [53] and other mammalian carnivores [32] can use to detect prey (e.g., a combination of visual, olfactory, and tactile). Therefore, where and when raccoons are more active may be a better determinant of predation risk than the type sensory cue provided by juvenile turtles.

Our findings suggest rodents may also be important predators of juvenile turtles. Rodent interactions did not vary by habitat type, suggesting they could be ubiquitous across habitats, or this could be due to the comparatively lower number of rodent interactions we documented. It is unclear why there was a higher likelihood of rodent interactions at FCTC compared to VRO and NHMW, but more rodents might occupy that site given it is much larger than the other two sites. Rodents were more likely to interact with models placed closer to woody structure, likely because chipmunks and squirrels frequently use features such as downed trees and brush piles [36]. Juvenile turtles occurring near these structures may be at higher risk of predation. Studies of other predator-prey systems suggest predation risk may be increased when prey select such habitat. For example, red-backed voles (*Clethrionomys gapperi*) selecting woody debris were at greater risk of predation by American marten (*Martes americana*) because this feature was thought to provide a sensory cue to enhance martens’ hunting success [54]. Also, small mammals occupying brush piles may be at greater risk of predation from western ratsnakes (*Pantherophis obsoletus*) [55]. Rodents were more likely to interact with exposed models rather than concealed ones and interacted with models predominantly during daylight hours, supporting the notion that visual cues are important for prey detection [22,23]. These results suggest juvenile turtle anti-predator behaviors such as hiding under cover may be more effective for reducing predation risk from rodents but less so for mesopredators.

The limited number of bird interactions we observed may be a function of the use of models rather than a reflection of the natural predator community. Avian predators generally rely on visual cues for prey detection [20,21]. Thus, incorporating movement into models may provoke greater predation attempts by birds beyond the simple visual cue of an exposed (albeit static) model [29,56] (but see [31,57]).

The secretive nature of juvenile turtles has largely precluded direct observations of predation under natural conditions, and monitoring 3D printed models with cameras could represent a feasible alternative. We acknowledge predators might have been attracted to models because of their novelty, particularly raccoons due to their curious nature. However, live juveniles are mobile and cryptic, so using models as proxies may currently be the most reasonable option. Our study has implications for conservation management of turtles and perhaps numerous terrestrial prey animals with similar body sizes, morphology, or behavior. Raccoons can be overpopulated in some areas due to human subsidies and reduction in top-order carnivores [58,59]. Habitat alteration, translocation, and predator control have been suggested as management actions to reduce the impacts of turtle predation [3,60,61]. Each of these tactics have merit, but they are not without shortcomings and have thus been subject to intense criticism [62,63,64]. Evaluating factors affecting predation risk as we did here could lead to improvements for efforts aimed at reducing encounters between juvenile turtles and their major predators.

## Figures and Tables

**Table 1:** Number of interactions (detection and attacks) with three-dimensional printed turtle models by each observed predator species.

Species	Detections	Attacks
Raccoon (*Procyon lotor*)	11	22
Virginia opossum (*Didelphis virginiana*)	2	2
Eastern chipmunk (*Tamias striatus*)	15	3
Eastern grey squirrel (*Sciurus carolinensis*)	1	0
Eastern fox squirrel (*Sciurus niger*)	4	1
Wild turkey (*Meleagris gallopavo*)	1	1
Unidentified small mammal	2	0

**Table 2:** Odds ratio estimates with 85% confidence intervals (CI) for predicting if predator groups (mesopredator or rodent) interacted with three-dimensional printed turtle models depending on numerous explanatory variables: study site (Nettie Hart Memorial Woods [NHMW] and Vermilion River Observatory [VRO] in relation to Fort Custer Training Center), habitat type (forest, grassland, and wetland in relation to forest edge), modal and maximum height (cm) of vegetation around models, distance (cm) from a model to woody structure, whether models were visually exposed in relation to concealed, whether models did not have turtle scent on them in relation to scented models, or the interaction of exposure and scent treatments.

**Mesopredator**	**Odds Ratio Estimate**	**85% CI**	
**NHMW**	**4.759**	**2.432**	**9.313**
VRO	1.785	0.816	3.907
forest	1.315	0.691	2.501
**grassland**	**0.156**	**0.032**	**0.755**
**wetland**	**2.351**	**1.064**	**5.193**
**modal vegetation height**	**0.962**	**0.938**	**0.986**
maximum vegetation height	0.996	0.985	1.006
distance from woody structure	1.001	0.999	1.003
exposure treatment	1.259	0.734	2.161
scent treatment	1.101	0.629	1.929
exposure and scent treatment interaction	1.664	0.568	4.878
**Rodent**	**Odds Ratio Estimate**	**85% CI**	
NHMW	0.655	0.285	1.508
**VRO**	**0.218**	**0.069**	**0.686**
forest	0.866	0.420	1.784
grassland	0.000	0.000	171.546
wetland	0.459	0.171	1.236
modal vegetation height	1.007	0.993	1.021
maximum vegetation height	0.992	0.981	1.003
**distance from woody structure**	**0.997**	**0.994**	**0.999**
**exposure treatment**	**2.871**	**1.440**	**5.727**
scent treatment	1.182	0.591	2.367
exposure and scent treatment interaction	2.199	0.550	8.795

Bolded values indicate confidence limits of odds ratio estimates excluding 1.

## Supplementary Materials

The following are available online at https://www.mdpi.com/2076-2615/10/2/275/s1, Video S1: Eastern chipmunk (*Tamias striatus*) in forest habitat biting an exposed three-dimensional printed turtle model with turtle scent applied to it. The concealed model can be seen in the right side of the frame between two sticks. Video S2: Eastern chipmunk in wetland habitat biting an exposed three-dimensional printed turtle model with turtle scent applied to it. Video S3: Virginia opossum (*Didelphis virginiana*) in forest habitat biting a concealed three-dimensional printed turtle model with turtle scent applied to it. The exposed model can be seen in the right side of the frame. Video S4: Raccoon (*Procyon lotor*) in forest habitat detecting a concealed three-dimensional printed turtle model with turtle scent applied to it. The exposed model can be seen in the right side of the frame. Video S5: Raccoon in forest habitat handling an exposed three-dimensional printed turtle model with turtle scent applied to it. Video S6: Raccoon in wetland habitat grasping and biting an exposed three-dimensional printed turtle model without turtle scent applied to it. Video S7: Raccoon in open habitat digging up a concealed three-dimensional printed turtle model without turtle scent applied to it. The exposed model can be seen in the left side of the frame. Video S8: Eastern fox squirrel (*Sciurus niger*) in forest habitat grasping and biting an exposed three-dimensional printed turtle model without turtle scent applied to it. Video S9: Eastern fox squirrel in edge habitat detecting an exposed three-dimensional printed turtle model without turtle scent applied to it. Video S10: Wild turkey (*Meleagris gallopavo*) in open habitat interacting with an exposed three-dimensional printed turtle model with turtle scent applied to it. Figure S1: Probability with 85% confidence interval of mesopredators and rodents interacting with three-dimensional printed turtle models (or no interaction) as a function of maximum vegetation height (cm) nearest to a model, Figure S2: Probability with 85% confidence interval of mesopredators and rodents interacting with three-dimensional printed turtle models (or no interaction) depending on whether models did or did not have juvenile box turtle scent cues applied to them, Table S1: Top-ranked multinomial regression models whose cumulative Akaike weight (w_i_) totaled 0.95 for predicting whether a three-dimensional printed turtle model would be interacted with by a particular predator group or not during a trial based on several explanatory variables. dist. wood = distance (cm) from a turtle model to woody structures such as log and slash piles; exposure = whether turtle models were visually exposed or concealed; habitat = forest, wetland, grassland, or edge; modal veg. = modal height (cm) of vegetation surrounding or nearest turtle models; max. veg. = maximum height (cm) of vegetation surrounding or nearest turtle models; site = study area (Fort Custer Training Center, Nettie Hart Memorial Woods, or Vermilion River Observatory); and scent = whether turtle models had turtle scent applied to them or not. ΔAICc is the difference in AICc for a given model from the minimum AICc. Table S2: Chemical compounds detected on cotton-tipped swabs using gas chromatography-mass spectrometry (details provided in Appendix A). Scented swabs were collected from captive-born juvenile eastern box turtles (*Terrapene carolina*). Unscented swabs were blank controls.

## Appendix A

To investigate whether we collected chemical compounds on scented swabs that predators could potentially use as an olfactory cue to detect scented models during field trials [24], we randomly selected three swab samples, each from a unique turtle, for analysis. We also analyzed two unscented (blank control) swabs to identify compounds on turtle scented swabs that were not detected on unscented swabs. Samples were analyzed using a gas chromatograph (Agilent model 6890, Agilent Technologies, Santa Clara, CA, USA) equipped with a BPX70 column connected to an Agilent 5973 mass-selective detector. Results from this analysis suggested we collected 34 unique compounds. Twenty-five of these compounds, which were predominantly fatty acid methyl esters and sterol derivatives, were detected only on scented swabs, whereas nine were detected on scented and unscented swabs (i.e., no unique compounds were found solely on unscented swabs; Table S2). Many of the compounds found on scented swabs are commonly detected as part of the skin lipid profile of vertebrates (e.g., [65,66]. Captive turtles were housed under an approved protocol (#16017) by the University of Illinois Institutional Animal Care and Use Committee and Scientific Collector’s Permits granted by the States of Michigan and Illinois (#NH17.5980).

## References

[1] Lovich J.E., Ennen J.R., Agha M., Gibbons J.W. (2018). Where have all the turtles gone, and why does it matter?. BioScience.

[2] Dodd C.K. (2001). North American Box Turtles: A Natural History.

[3] Browne C.L., Hecnar S.J. (2007). Species loss and shifting population structure of freshwater turtles despite habitat protection. Biol. Conserv..

[4] Oddie M.A.Y., Coombes S.M., Davy C.M. (2015). Investigation of cues used by predators to detect snapping turtle (*Chelydra serpentina*) nests. Can. J. Zool..

[5] Heithaus M.R., Wirsing A.J., Thomson J.A., Burkholder D.A. (2008). A review of lethal and non-lethal effects of predators on adult marine turtles. J. Exp. Mar. Biol. Ecol..

[6] Sinclair A.R.E., Pech R.P., Dickman C.R., Hik D., Mahon P., Newsome A.E. (1998). Predicting effects of predation on conservation of endangered prey. Conserv. Biol..

[7] Ernst C.H., Lovich J.E., Barbour R.W. (1994). Turtles of the United States and Canada.

[8] Fincham J.E., Lambrechts N. (2014). How many tortoises do a pair of pied crows (*Corvus albus*) need to kill to feed their chicks?. Ornithol. Obs..

[9] Nagy K.A., Hillard L.S., Tuma M.W., Morafka D.J. (2015). Head-started desert tortoises (*Gopherus agassizii*): Movements, survivorship and mortality causes following their release. Herpetol. Conserv. Biol..

[10] Tetzlaff S.J., Sperry J.H., DeGregorio B.A. (2018). Attempted depredation of a juvenile Eastern Box Turtle, *Terrapene carolina* (Linnaeus, 1758), by Sandhill Cranes, *Antigone canadensis* (Linnaeus, 1758). Herpetol. Notes.

[11] Callahan J.R. (1993). Squirrels as predators. Great Basin Nat..

[12] Belzer W.R., Seibert S., Atkinson B. (2000). Putative chipmunk predation of juvenile eastern box turtles. Turt. Tortoise Newsl..

[13] Jones M.T., Sievert P.R. (2012). Elevated mortality of hatchling Blanding’s turtles (*Emydoidea blandingii*) in residential landscapes. Herpetol. Conserv. Biol..

[14] Pike D.A., Pizzatto L., Pike B.A., Shine R. (2008). Estimating survival rates of uncatchable animals: The myth of high juvenile mortality in reptiles. Ecology.

[15] Arsovski D., Tomović L., Golubović A., Nikolić S., Sterijovski B., Ajtić R., Ballouard J., Bonnet X. (2018). When carapace governs size: Variation among age classes and individuals in a free-ranging ectotherm with delayed maturity. Oecologia.

[16] Paterson J.E., Steinberg B.D., Litzgus J.D. (2012). Revealing a cryptic life-history stage: Differences in habitat selection and survivorship between hatchlings of two turtle species at risk (*Glyptemys insculpta* and *Emydoidea blandingii*). Wildl. Res..

[17] Lima S.L., Bednekoff P.A. (1999). Temporal variation in danger drives antipredator behavior: The predation risk allocation hypothesis. Am. Nat..

[18] Atwood T.C., Gese E.M., Kunkel K.E. (2009). Spatial partitioning of predation risk in a multiple predator-multiple prey system. J. Wildl. Manag..

[19] Thaker M., Vanak A.T., Owen C.R., Ogden M.B., Niemann S.M., Slotow R. (2011). Minimizing predation risk in a landscape of multiple predators: Effects on the spatial distribution of African ungulates. Ecology.

[20] Remsen J.V., Robinson S.K. (1990). A classification scheme for foraging behavior of birds in terrestrial habitats. Stud. Avian Biol..

[21] Santisteban L., Sieving K.E., Avery M.L. (2002). Use of sensory cues by fish crows *Corvus ossifragus* preying on artificial bird nests. J. Avian Biol..

[22] McQuade D.B., Williams E.H., Eichenbaum H.B. (1986). Cues used for localizing food by the gray squirrel (*Sciurus carolinensis*). Ethology.

[23] Duncan R.D., Jenkins S.H. (1998). Use of visual cues in foraging by a diurnal herbivore, Belding’s ground squirrel. Can. J. Zool..

[24] Conover M.R. (2007). Predator–Prey Dynamics, the Role of Olfaction.

[25] Fogarty D.T., Elmore R.D., Fuhlendorf S.D., Loss S.R. (2018). Variation and drivers of airflow patterns associated with olfactory concealment and habitat selection. Ecology.

[26] Blomberg S., Shine R., Sutherland W. (2004). Reptiles. Practical Census Techniques for Animal Populations.

[27] Bateman P.W., Fleming P.A., Wolfe A.K. (2017). A different kind of ecological modelling: The use of clay model organisms to explore predator–prey interactions in vertebrates. J. Zool..

[28] Behm J.E., Waite B.R., Hsieh S.T., Helmus M.R. (2018). Benefits and limitations of three-dimensional printing technology for ecological research. BMC Ecol..

[29] Walker M., Humphries S. (2019). 3D Printing: Applications in evolution and ecology. Ecol. Evol..

[30] Akcali C.K., Pérez-Mendoza H.A., Salazar-Valenzuela D., Kikuchi D.W., Guayasamin J.M., Pfennig D.W. (2019). Evaluating the utility of camera traps in field studies of predation. PeerJ.

[31] Hansen N.A., Sato C.F., Michael D.R., Lindenmayer D.B., Driscoll D.A. (2019). Predation risk for reptiles is highest at remnant edges in agricultural landscapes. J. Appl. Ecol..

[32] Lawson R.R., Fogarty D.T., Loss S.R. (2019). Use of visual and olfactory sensory cues by an apex predator in deciduous forests. Can. J. Zool..

[33] Van Dijk P.P. Terrapene Carolina (Errata Version Published in 2016). The IUCN Red List of Threatened Species 2011. https://www.iucn.org/sites/dev/files/import/downloads/arabian_oryx_factsheet.pdf.

[34] Budischak S.A., Hester J.M., Price S.J., Dorcas M.E. (2006). Natural history of *Terrapene carolina* (Box Turtles) in an urbanized landscape. Southeast. Nat..

[35] Tetzlaff S.J., Sperry J.H., Kingsbury B.A., DeGregorio B.A. (2019). Captive-rearing duration may be more important than environmental enrichment for enhancing turtle head-starting success. Glob. Ecol. Conserv..

[36] Blouin-Demers G., Weatherhead P.J. (2001). Habitat use by black rat snakes (*Elaphe obsoleta obsoleta*) in fragmented forests. Ecology.

[37] Ryan D.A., Larson J.S. (1976). Chipmunks in residential environments. Urban Ecol..

[38] Sato C.F., Wood J.T., Schroder M., Green K., Osborne W.S., Michael D.R., Lindenmayer D.B. (2014). An experiment to test key hypotheses of the drivers of reptile distribution in subalpine ski resorts. J. Appl. Ecol..

[39] R Core Team (2017). R: A Language and Environment for Statistical Computing.

[40] Venables W.N., Ripley B.D. (2002). Modern Applied Statistics with S.

[41] Barton K. (2018). MuMIn: Multi-Model Inference. R Package Version 1.42.1. https://CRAN.R-project.org/package=MuMIn.

[42] Akaike H., Petrov B.N., Csaki B.F. (1973). Information theory and an extension of the maximum likelihood principle. Second International Symposium on Information Theory.

[43] Symonds M.R.E., Moussalli A. (2011). A brief guide to model selection, multimodel inference and model averaging in behavioural ecology using Akaike’s information criterion. Behav. Ecol. Sociobiol..

[44] Arnold T.W. (2010). Uninformative parameters and model selection using Akaike’s Information Criterion. J. Wildl. Manag..

[45] Tucker C.R., Strickland J.T., Edmond B.S., Delaney D.K., Ligon D.B. (2015). Activity patterns of ornate box turtles (*Terrapene ornata*) in northwestern Illinois. Copeia.

[46] Fritzell E.K. (1978). Habitat use by prairie raccoons during the waterfowl breeding season. J. Wildl. Manag..

[47] Pedlar J.H., Fahrig L., Merriam H.G. (1997). Raccoon habitat use at 2 spatial scales. J. Wildl. Manag..

[48] Kamler J.F., Gipson P.S. (2003). Space and habitat use by male and female raccoon, *Procyon lotor*, in Kansas. Can. Field Nat..

[49] Barding E.E., Nelson T.A. (2008). Raccoons use habitat edges in northern Illinois. Am. Midl. Nat..

[50] Frey S.N., Conover M.R. (2006). Habitat use by meso-predators in a corridor environment. J. Wildl. Manag..

[51] DeGregorio B.A., Weatherhead P.J., Sperry J.H. (2014). Power lines, roads, and avian nest survival: Effects on predator identity and predation intensity. Ecol. Evol..

[52] Anderson L., Burgin S. (2008). Patterns of bird predation on reptiles in small woodland remnant edges in peri-urban north-western Sydney, Australia. Landsc. Ecol..

[53] Bowman G.B., Harris L.D. (1980). Effect of spatial heterogeneity on ground-nest depredation. J. Wildl. Manag..

[54] Andruskiw M., Fryxell J.M., Thompson I.D., Baker J.A. (2008). Habitat-mediated variation in predation risk by the American marten. Ecology.

[55] Sperry J.H., Weatherhead P.J. (2010). Ratsnakes and brush piles: Intended and unintended consequences of improving habitat for wildlife?. Am. Midl. Nat..

[56] Paluh D.J., Hantak M.M., Saporito R.A. (2014). A test of aposematism in the dendrobatid poison frog *Oophaga pumilio*: The importance of movement in clay model experiments. J. Herpetol..

[57] Webb J.K., Whiting M.J. (2005). Why don’t small snakes bask? Juvenile broad-headed snakes trade thermal benefits for safety. Oikos.

[58] Garrott R.A., White P.J., Vanderbilt White C.A. (1993). Overabundance: An issue for conservation biologists?. Conserv. Biol..

[59] Crooks K.R., Soule M.E. (1999). Mesopredator release and avifaunal extinctions in a fragmented system. Nature.

[60] Boarman W.I. (2003). Managing a subsidized predator population: Reducing common raven predation on desert tortoises. Environ. Manag..

[61] Spencer R.J., Van Dyke J.U., Thompson M.B. (2017). Critically evaluating best management practices for preventing freshwater turtle extinctions. Conserv. Biol..

[62] Burke R.L. (2015). Head-starting turtles: Learning from experience. Herpetol. Conserv. Biol..

[63] Treves A., Krofel M., McManus J. (2016). Predator control should not be a shot in the dark. Front. Ecol. Environ..

[64] Taylor G., Canessa S., Clarke R.H., Ingwersen D., Armstrong D.P., Seddon P.J., Ewen J.G. (2017). Is reintroduction biology an effective applied science?. TREE.

[65] Khannoon E.R., Lunt D.H., Schulz S., Hardege J.D. (2013). Divergence of scent pheromones in allopatric populations of *Acanthodactylus boskianus* (Squamata: Lacertidae). Zool. Sci..

[66] Pannkuk E.L., Fuller N.W., Moore P.R., Gilmore D.F., Savary B.J., Risch T.S. (2014). Fatty acid methyl ester profiles of bat wing surface lipids. Lipids.

