# Supplementary material for: Identification of Factors Affecting Predation Risk for Juvenile Turtles Using 3D Printed Models
Source: Animals (Basel). 2020 Feb 11;10(2):275. doi: 10.3390/ani10020275 (PMC7070983; doi:10.3390/ani10020275)
Supplement: Supplementary file 1 [file animals-10-00275-s001.pdf]

Supplementary Information

Identification of Factors Affecting Predation Risk for Juvenile Turtles using 3D Printed Models

Sasha J. Tetzlaff [1,2,*], Alondra Estrada [1], Brett A. DeGregorio [3] and Jinelle H. Sperry [1,2]

[1] Department of Natural Resources and Environmental Sciences, University of Illinois at Urbana-Champaign, Urbana, IL 61801, USA; alondra4@illinois.edu (A.E.); Jinelle.Sperry@usace.army.mil (J.H.S.)
[2] US Army Construction Engineering Research Laboratory, Champaign, IL 61822, USA
[3] U.S. Geological Survey, University of Arkansas Fish and Wildlife Cooperative Research Unit, Fayetteville, AR 72701, USA; Bdegrego@uark.edu
* Correspondence: sashatetzlaff@gmail.com

Supplemental videos

Video S1: Eastern chipmunk (*Tamias striatus*) in forest habitat biting an exposed three-dimensional printed turtle model with turtle scent applied to it. The concealed model can be seen in the right side of the frame between two sticks. Video link.

Video S2: Eastern chipmunk in wetland habitat biting an exposed three-dimensional printed turtle model with turtle scent applied to it. Video link.

Video S3: Virginia opossum (*Didelphis virginiana*) in forest habitat biting a concealed three-dimensional printed turtle model with turtle scent applied to it. The exposed model can be seen in the right side of the frame. Video link.

Video S4: Raccoon (*Procyon lotor*) in forest habitat detecting a concealed three-dimensional printed turtle model with turtle scent applied to it. The exposed model can be seen in the right side of the frame. Video link.

Video S5: Raccoon in forest habitat handling an exposed three-dimensional printed turtle model with turtle scent applied to it. Video link.

Video S6: Raccoon in wetland habitat grasping and biting an exposed three-dimensional printed turtle model without turtle scent applied to it. Video link.

Video S7: Raccoon in open habitat digging up a concealed three-dimensional printed turtle model without turtle scent applied to it. The exposed model can be seen in the left side of the frame. Video link.

Video S8: Eastern fox squirrel (*Sciurus niger*) in forest habitat grasping and biting an exposed three-dimensional printed turtle model without turtle scent applied to it. Video link.

Video S9: Eastern fox squirrel in edge habitat detecting an exposed three-dimensional printed turtle model without turtle scent applied to it. Video link.

Video S10: Wild turkey (*Meleagris gallopavo*) in open habitat interacting with an exposed three-dimensional printed turtle model with turtle scent applied to it. Video link.

Supplemental figures and tables

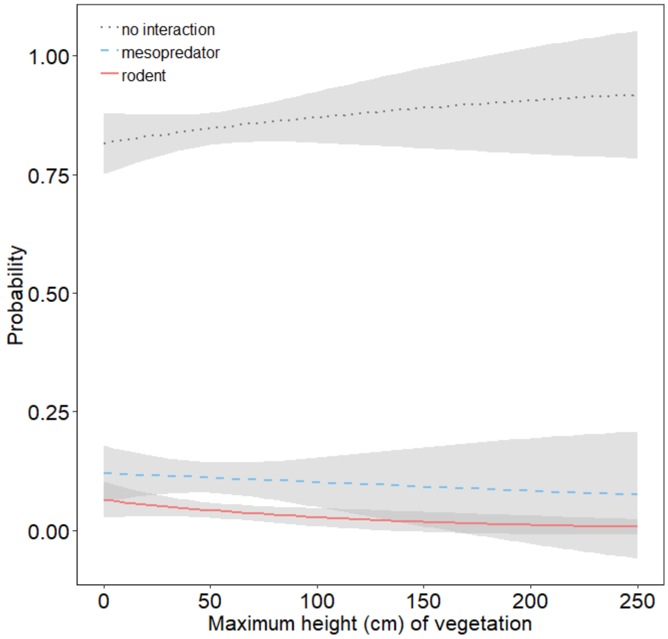

Figure S1: Probability with 85% confidence interval of mesopredators and rodents interacting with three-dimensional printed turtle models (or no interaction) as a function of maximum vegetation height (cm) nearest to a model.

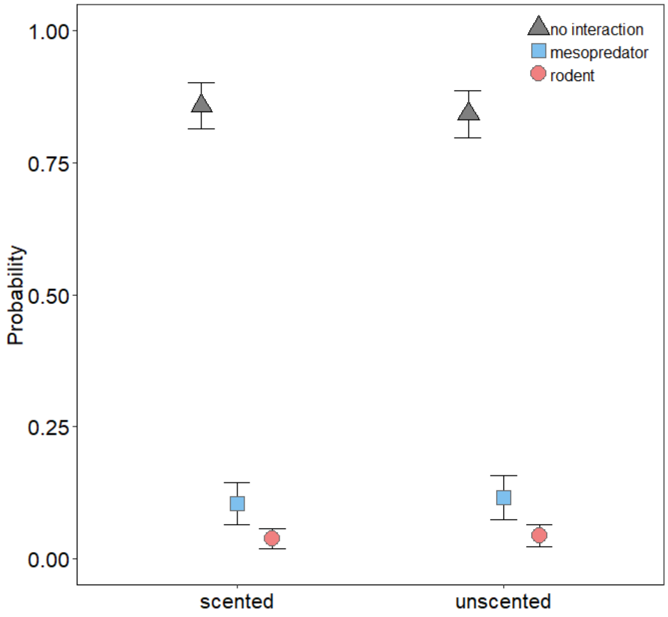

Figure S2: Probability with 85% confidence interval of mesopredators and rodents interacting with three-dimensional printed turtle models (or no interaction) depending on whether models did or did not have juvenile box turtle scent cues applied to them.

Table S1: Top-ranked multinomial regression models whose cumulative Akaike weight (w_i) totaled 0.95 for predicting whether a three-dimensional printed turtle model would be interacted with by a particular predator group or not during a trial based on several explanatory variables. dist. wood = distance (cm) from a turtle model to woody structures such as log and slash piles; exposure = whether turtle models were visually exposed or concealed; habitat = forest, wetland, grassland, or edge; modal veg. = modal height (cm) of vegetation surrounding or nearest turtle models; max. veg. = maximum height (cm) of vegetation surrounding or nearest turtle models; site = study area (Fort Custer Training Center, Nettie Hart Memorial Woods, or Vermilion River Observatory); and scent = whether turtle models had turtle scent applied to them or not. ΔAICc is the difference in AICc for a given model from the minimum AICc.

Model	ΔAIC_c	w_i	Log-likelihood
dist. wood + exposure + habitat + modal veg. + site	0.000	0.228	-172.120
exposure + habitat + modal veg. + site	0.787	0.154	-174.718
dist. wood + habitat + modal veg. + site	1.277	0.121	-174.964
habitat + modal veg. + site	1.824	0.092	-177.417
dist. wood + exposure + habitat + modal veg. + site + max. veg.	2.537	0.064	-171.158
exposure + habitat + site	3.634	0.037	-178.322
exposure + habitat + modal veg. + site + max. veg.	3.821	0.034	-174.031
dist. wood + habitat + modal veg. + site + max. veg.	3.921	0.032	-174.080
dist. wood + exposure + habitat + modal veg. + site + scent	4.163	0.028	-171.971
dist. wood + exposure + habitat + site	4.607	0.023	-176.628
habitat + site	4.721	0.022	-181.021
habitat + modal veg. + site + max. veg.	4.908	0.020	-176.779
exposure + habitat + modal veg. + site + scent	4.939	0.019	-174.590
exposure + habitat + site + max. veg.	5.209	0.017	-176.929
dist. wood + habitat + modal veg.+ site + scent	5.395	0.015	-174.818
habitat + modal veg. + site + scent	5.932	0.012	-177.291
dist. wood + exposure + modal veg. + site	5.947	0.012	-181.634
dist. wood + habitat + site	5.964	0.012	-179.487
dist. wood + exposure + habitat + site + max.veg	6.131	0.011	-175.186

Table 2. Chemical compounds detected on cotton-tipped swabs using gas chromatography-mass spectrometry (details provided in Appendix A). Scented swabs were collected from captive-born juvenile eastern box turtles (*Terrapene carolina*). Unscented swabs were blank controls.

Compound
**Detected only on scented swabs**
11-EICOSANOIC ACID, METHYL ESTER
13-DOCOSENOIC ACID, METHYL ESTER, (Z)-
17-(1,5-DIMETHYLHEXYL-10,13-DIMETHYL-2,3,4,7,8,9,10,11,12,13,14,15,16,17-TETRADECAHYDRO-1H-CYCLOPENTA[A]PHENANTHREN-3-OL
8-OCTADECANOIC ACID, METHYL ESTER
9, 12-OCTADECADIENOIC ACID, (Z,Z)-, METHYL ESTER
9-HEXADECANOIC ACID, METHYL ESTER, (Z)-
BENZOIC ACID, METHYL ESTER
CHOLESTAN-3-ONE
CYCLOPROPANEOCTANOIC ACID, 2-OCTYL-, METHYL ESTER, CIS-
DIETHYLTOLUAMIDE
DOCOSANOIC ACID, 2-HYDROXY-, METHYL ESTER
DODECANOIC ACID, METHYL ESTER
HENEICOSANOIC ACID, METHYL ESTER

Table S2. *Cont.*

HEPTAQDECANOIC ACID, METHYL ESTER
HEXACOSANOIC ACID, METHYL ESTER
HEXADECANOIC ACID, METHYL ESTER
METHYL 9-METHYLTETRADECANOATE
METHYL TETRADECANOATE
NONANEDIOIC ACID, DIMETHYL ESTER
OCTACOSANOIC ACID, METHYL ESTER
PENTACOSANOIC ACID, METHYL ESTER
PENTADECANOIC ACID, METHYL ESTER
SIGMASTAN-3,5-DIENE
TRIACONTANOIC ACID, METHYL ESTER
TRICOSANOIC ACID, METHYL ESTER
**Detected on scented and unscented swabs**
8,11-OCTADECADIENOIC ACID, METHYL ESTER
9-OCTADECANOIC ACID, METHYL ESTER, (E)-
DOCOSANOIC ACID, METHYL ESTER
EICOSANOIC ACID, METHYL ESTER
HEPTADECANOIC ACID, METHYL ESTER
HEXADECANOIC ACID, METHYL ESTER
OCTADECANOIC ACID, METHYL ESTER
PENTADECANOIC ACID, 14-METHYL-, METHYL ESTER
TETRACOSANOIC ACID, METHYL ESTER
